# MiR-451a and let-7i-5p loaded extracellular vesicles attenuate heme-induced inflammation in hiPSC-derived endothelial cells

**DOI:** 10.3389/fimmu.2022.1082414

**Published:** 2022-12-22

**Authors:** Justin J. Thomas, Keri Oxendine Harp, Alaijah Bashi, Joshua L. Hood, Felix Botchway, Michael D. Wilson, Winston E. Thompson, Jonathan K. Stiles, Adel Driss

**Affiliations:** ^1^ Department of Physiology, Morehouse School of Medicine, Atlanta, GA, United States; ^2^ Department of Pharmacology and Toxicology, Brown Cancer Center, Hepatobiology and Toxicology COBRE, University of Louisville School of Medicine, Louisville, KY, United States; ^3^ Department of Pathology, Korle-Bu Teaching Hospital, University of Ghana Medical School, Accra, Ghana; ^4^ Department of Parasitology, Noguchi Memorial Institute for Medical Research, University of Ghana, Accra, Ghana; ^5^ Department of Microbiology, Biochemistry, and Immunology, Morehouse School of Medicine, Atlanta, GA, United States

**Keywords:** malaria, sickle cell anemia, extracellular vesicles, micro-RNA, hiPSC, inflammation, exosomes, liposomes

## Abstract

Hemolysis is associated with many pathologies, including trauma, sepsis, hemorrhagic stroke, malaria, and genetic disorders such as sickle cell disease (SCD). When hemolysis occurs, free-heme drives vascular inflammation, resulting in oxidative tissue damage and cardiometabolic complications. A better understanding of heme clearance and detoxification is essential to preventing sustained tissue damage. Human induced pluripotent stem cell (hiPSC)-derived endothelial cells (hiPSC-ECs) provide a novel source of patient-specific cells and tissues for disease modeling, drug discovery, and regenerative therapeutics. Here we report the use of hiPSC-ECs to elucidate the role of miR-451a and let-7i-5p-loaded extracellular vesicles (EVs, such as exosomes) in the inflammatory response to free-heme as a model for heme-induced inflammation. We provide evidence of a significant correlation between miR-451a and let-7i-5p-loaded circulating exosomes in *plasmodium*-infected patients with reported clinical benchmarks of malaria-severity (e.g., Hemoglobin (Hb) levels, white blood cell counts). Additionally, we determined that exposure of *Plasmodium falciparum* (*Pf*) parasites to EVs, loaded with either miRNA, significantly reduces their counts *in vitro.* Using hiPSCs derived from individuals with wild-type Hb (HbAA) or homozygous sickle cell mutated Hb (HbSS) genotypes, we demonstrate that heme-treated hiPSC-ECs secreted inflammatory products (cytokines, chemokines and growth factors) into supporting media at concentrations that were similar to that reported in HbAA and HbSS serum. This inflammatory response was attenuated by exposure with miR-451a or let-7i-5p-loaded EVs. We also found a decrease in transcription of ICAM1 and P-Selectin, as well as the secretion of key inflammatory cytokines (e.g., CXCL10, TNF-α, and IFN-γ). Based on these findings, we propose a model in which increased levels of exosomal miR-451a and let-7i-5p in *Plasmodium*-infected individuals will attenuate inflammatory responses to free-heme and parasite-derived products. As a result, infected erythrocytes will less likely adhere to the endothelium, sequester in brain micro vessels, and reduce vaso-occlusive crises that exacerbate cerebral malaria.

## Introduction

More than half a million people died of malaria in 2020, primarily in children under five years of age on the African continent (1). Of the *Plasmodium* parasites, *Plasmodium falciparum* (*Pf*) parasite is responsible for the highest mortality rate and severe illness associated with malaria (2, 3). *Pf* infected red blood cells (iRBCs) induce cytoadherence, which increases clinical severity (4, 5). Postmortem studies reveal that cytoadherence by iRBCs to endothelial cells lead to a variety of pathological sequelae, including vaso-occlusive crises and petechial hemorrhaging (e.g., cerebral malaria) (6, 7). Additionally, the severity of *Pf* infection may vary based on the robustness of host immunity, and genetic factors (8). For instance, people with the sickle cell trait (SCT), who carry a hemoglobin (Hb) gene with HbAS genotype (in which one abnormal allele (HbS) and one normal allele (HbA) are present), tend to have lower malaria mortality rates (8–10). In contrast, people with sickle cell anemia (SCA), homozygous HbSS, are more likely to die of malaria than those with wildtype (normal) Hb (homozygous HbAA) or those with SCT (10). Furthermore, SCA patients are more likely to develop severe malaria symptoms compared to non-SCA patients (9, 11). Patients with SCA often have more severe malaria due to their shorter RBC lifespan, as well as the parasite rupturing them, releasing higher levels of toxic heme into the body (12–14). A better understanding of how malaria interact with sickle cell disease (SCD) will allow for the development of novel interventions to reduce the severity of both diseases.

Previous studies have identified specific microRNA (miRNA) in circulation, including in extracellular vesicles (EVs, such as exosomes), isolated from *Plasmodium* parasite-infected patients which have been identified as potential mediators of malaria severity (15–19). Recent studies have examined the numerous roles that EVs play in malaria. An infection with a severe disease can raise EVs from various types of cells, including endothelial cells, RBCs, and platelets. As a result, EVs might contribute to inflammation and malaria pathogenesis. EVs are released by iRBCs as the parasites differentiate into them (20, 21). Also, miRNAs in EVs have been shown to alter gene expression of endothelial cells cultured *in vitro*, suggesting that they may play a role in malaria pathophysiology (22, 23). In a recent study, we reported that two miRNAs, miR-451a and let-7i-5p, were differentially accumulated in the circulating EVs of parasite-infected individuals, based on their sickle cell Hb sickle genotypes (18). *Plasmodium* parasite-infected HbSS patients have significantly lower levels of miR-451a in circulating EVs compared to levels in parasite-infected individuals who are HbAA or HbAS genotypes. Finally, parasite-infected HbSS individuals had lower levels of circulating EV-miR-451a and let-7i-5p compared to non-infected HbSS individuals (18).

Both miR-451 and the let-7 family of miRNAs regulate genes involved in inflammation (24–26). MiR-451a also plays a crucial role in the differentiation and oxidative stress of erythrocytes (26). Let-7i-5p directly inhibits the 3′-UTR of the BACH1 (BTB Domain and CNC Homolog 1) protein, resulting in increased expression of heme-oxygenase-1 (HO-1), a key enzyme that degrades heme groups (26–28). Increased levels of free-heme caused by RBC-hemolysis during *Plasmodium* parasite infection and SCD lead to inflammation, which damages the host’s vascular endothelium and ultimately exacerbates crisis during malaria and SCD pathogenesis (29–32). Furthermore, increased circulation of free-heme mediates malaria severity, specifically cerebral malaria (33, 34). Considering these findings, we hypothesized that miR-451a and let-7i-5p may have critical roles in the pathogenesis of malaria.

Data from malaria patients in the Accra region of Ghana, West Africa, revealed physiological and molecular markers that correlated with the EV expression of miR-451a and let-7i-5p (18, 35). We developed an *in vitro* endothelial cell (EC) hemolytic model using human-induced pluripotent stem cells (hiPSCs) derived from uninfected HbAA and HbSS individuals that recapitulates the inflammatory response to heme observed in both SCD and malaria-positive individuals. The hiPSC-EC provides a patient-specific cell lines for disease modeling and miRNA functional studies. Furthermore, we confirmed that miR-451a and let-7i-5p-loaded in synthetically derived lipid-based EVs (i.e., liposomes) target inflammatory regulators simultaneously, suggesting potential use of those miRNAs, loaded in EVs, for attenuation of malaria-associated inflammation.

## Materials and methods

### Cultivation of Pf

**Figure 1:**
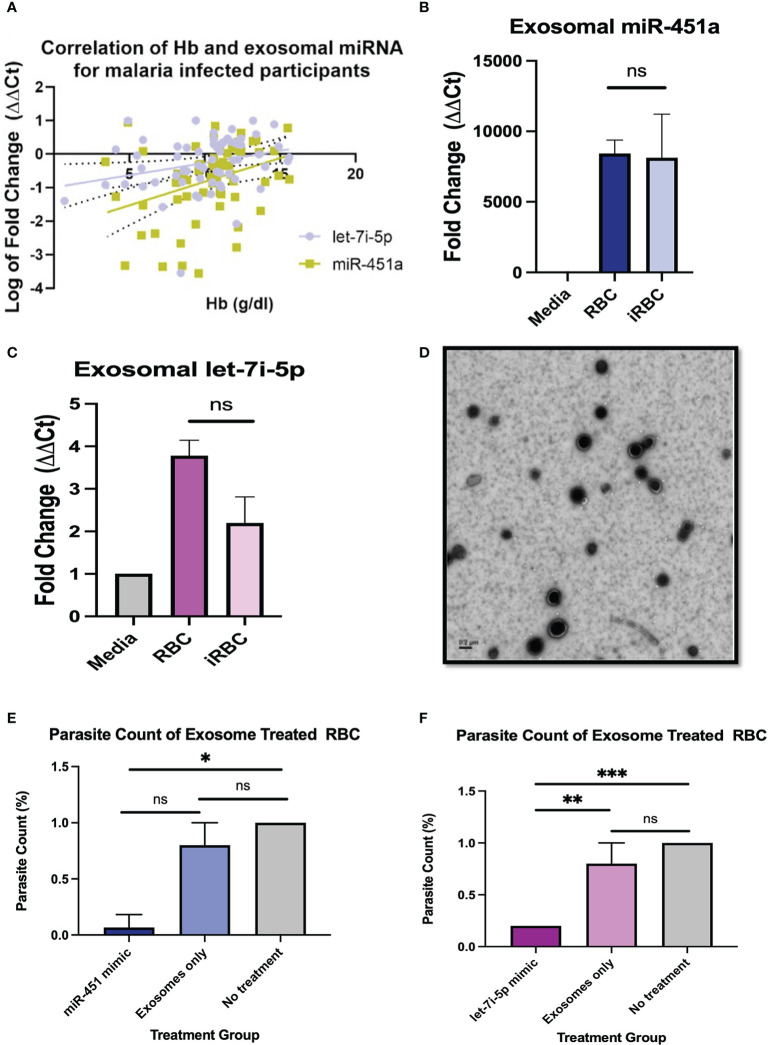
Pathophysiology of miRNA-loaded EVs in *P. Falciparum* infection **(A)** Pearson correlation between exosomal miR-451a and let-7i-5p with Hb resulted in R^2 =^ 0.12 p<0.01, and R^2 =^ 0.07, P<0.05, respectively. **(B, C)** Exosomal RNA was isolated from cell media without cells, supernatant from RBCs, and infected RBCs (iRBC). RT-qPCR analysis of miR-451a and let-7i-5p was performed. CT values were normalized to cell media without cells and using U6 internal control. Significance was analyzed using unpaired t-test. No significant difference of exosomal miRNA was found between RBCs and iRBCs when normalized to media. **(D)** Negative stain electron microscopy of exosomes isolated form RBCs. Scale is 0.2µm. **(E, F)** Cells were treated with 8.25 x 10^11^ exosome in 100µL with a dilution of 1:5 (exosomes: media). Graphs indicate parasite count percentage for exosomes miR-451a or let-7i-5p mimic exosomes with nothing encapsulated or iRBC not treated with any exosomes (N = 3 to 6 experiments per treatment). Treatment of exosomes containing miR-451a mimic significantly reduced (P=0.02) parasite count compared to no treatment. Treatment of exosomes containing let-7i-5p mimic significantly (P<0.0001) reduced parasite count compared to not treatment. ns indicates no significant difference. Significance was analyzed using unpaired t-test. * indicates p < 0.05, ** indicates p < 0.01, *** indicates p < 0.001 for all bar graphs.

**Figure 2:**
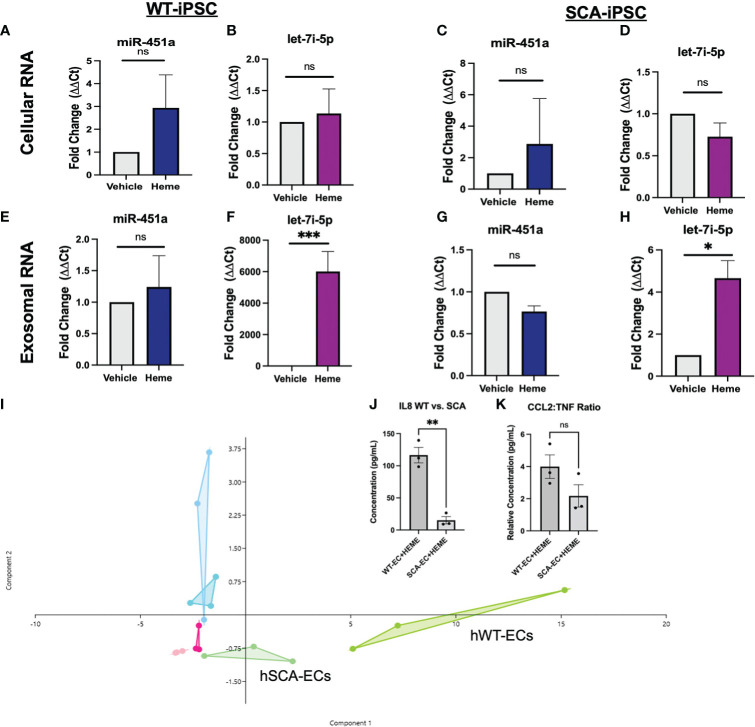
Molecular Effects of Heme-Induced Inflammation on Endothelial Cells **(A–D)** RT-qPCR of total lysate mRNA expression of miR-451a and let-7i-5p in respective hiPSC-ECs treated with either DMSO vehicle of 30 μM or Heme for 24 hrs. Paired t-test was performed for statistical analysis (n=3) **(E–H)** RT-qPCR of exosomal mir-451a and let-7i-5p expression in respective hiPSC-ECs treated with either DMSO vehicle or 30 μM Heme for 24 hrs. Paired-t test was performed for statistical analysis. Paired t-test was performed for statistical analysis **(I)**. Overlapping Principal Component Analysis of cytokine inflammatory profile (representing 26 cytokines) of hiPSC-ECs treated with DMSO vehicle, 30 μM Heme or untreated cells of either WT or SCA hemoglobin genotypes. Cytokine ratios were normalized to basic FGF value. The Aqua color represents the WT-EC groups. The dark pink represents the WT-EC-vehicle groups. The chartreuse figure represents the WT-EC and Heme treatment groups. The light blue represents the SCA-EC only groups. The light pink represents the SCA-EC-vehicle group. The light green represents the SCA-EC-Heme treatment groups (hSCA-ECs). The component 1 and component 2 eigenvalue and percent variance are 22.7 and 87.6% and 1.6 and 6.2%, respectively. **(J)** Comparison of absolution IL-8 concentration between WT-ECs and SCA-ECs treated with 30 μM Heme for 24 hrs. Un-paired t-test was performed for statistical analysis (n=3) **(K)** Comparison of CCL2:TNF-alpha cytokine ratio in WT-ECs and SCA-ECs treated with 30 μM of Heme for 24 hrs (n=3). Unpaired t-test was performed for statistical analysis. ns indicates no significant difference, * indicates p < 0.05, ** indicates p < 0.01, *** indicates p < 0.001 for all bar graphs.

*Pf* strain 3D7 (MRA-102) was obtained from the BEI Resources (managed by the American Type Culture Collection ATCC, Manassas, VA, USA). Pathogen-free and non-SCD O+ whole blood was purchased from Interstate Blood Bank (Memphis, Tennessee, USA). RBCs were isolated from whole blood and used in the parasite culture. *Pf* were cultured following the WHO-FIND-CDC Malaria RDT Product Testing Methods Manual (Version 7)-2018 SOP 4.1 Preparation of Reagents and Media for Culture of Malaria Parasites (36). Human serum was substituted by AlbuMAX (Cat# 11021029, Gibco/ThermoFisher Scientific) in culture media for experiments where iRBCs were treated with miRNA loaded exosomes, as it has been widely used to culture *Pf*, given the lack of exosomes present in the serum substitute (37). All parasites were cultured using 10% hematocrit (Hct) for general culture and 2% Hct for experimental assays. Culture flasks were incubated at 37°C with a special gas mixture of 5% CO_2_, 5% O_2_, and 90% N_2_. *Pf* NF54E (BEI Resources, MRA-1000) was obtained from John Hopkins, cultured at 4% Hct, and had supernatant collection from iRBCs with different parasitemia. *Pf* NF54 was cultured using pooled serum but using the same protocol as the *Pf* 3D7. Experiments using *Pf* NF54 is presented in **Figures 1B, C**; parasitemia of experiment ranged from 0.6% to 6.1%. 3D7 is a subclone of NF54 and both share same pathological characteristics (38). All parasite culture experiments used *Pf* 3D7 except for data presented in **Figures 2B, C**. Parasite counts were determined using thick smear microscopy, the gold standard method in malaria diagnosis labs. In brief, each sample of iRBCs was smeared on a slide, methanol-fixed, air dried and stained with 2.5% Giemsa stain, which specifically stains parasites. For each slide, at least 10 random fields of view are analysed and the number of infected RBCs are divided by the total number of RBCs counted.

### Collection of plasmodium parasite-infected plasma samples

As previously reported, institutional review boards ethical reviews were obtained from respective collaborator institutions, including Morehouse School of Medicine (MSM, Atlanta, GA), University of Ghana College of Health Sciences in the Korle-Bu teaching hospital, and Noguchi Memorial Institute for Medical Research (39). Briefly, written consent from participants were obtained from individuals living in the greater Accra region in Ghana, West Africa. Individuals included both sexes with and without malaria. The cohort consisted of a total of 64 individuals with an average age of 28.5 18.9 collected in 2018-2019. Written consent was provided by parents of participants under the age of 18. To anonymize the data, each participant was given a numerical code. Individuals were excluded if they were pregnant, HIV positive, abnormal fetal Hb (HbF), or lacking complete blood count (CBC) count information. Blood samples were collected and prepared for analysis as previously described (39).

### Exosome isolation and miRNA nucleofection

Patient plasma exosomes for the population-based cohort study were isolated according to manufacturer’s instructions using Invitrogen’s Total Exosome Isolation kit from plasma (Invitrogen, cat# 4484450). Briefly, frozen plasma samples were thawed and centrifuged at 2,000 x g for 20 minutes. Supernatant was collected and transferred to a new tube and subsequently centrifuged at 10,000 x g for 20 minutes. The supernatant was then collected and 0.5 volume of 1X PBS was added to 0.2 volume of Exosome Precipitation Reagent (from plasma) and vortexed. Samples were then incubated at room temperature for 10 minutes followed by centrifugation at 10,000 x g for 5 minutes at room temperature. The supernatants were discarded, and exosomes were resuspended in 1X PBS.

For exosomes used for *in vitro* studies, RBCs were isolated from whole blood and washed 3-times with incomplete RPMI media (iRPMI) (**Figure 1E-F**). A 50% Hct solution was created in iRPMI, and flasks were incubated overnight at 37°C. Resultant supernatant was collected and centrifuged at 600 x g for 20 minutes at 4°C. Supernatant was then collected and centrifuged at 3260 x g for 15 minutes at 4°C. Again, supernatant was collected and centrifuged at 10,000 x g for 30 minutes at 4°C. The following supernatant was then collected and ultracentrifuged at 100,000 x g for 70 minutes at 4°C. After ultracentrifugation, supernatant was collected and stored at -20°C until electroporation. Exosome isolation was confirmed by NanoSight analysis at the MSM’s Microvesicle Core.

RBC’s exosomes were loaded with miRNA mimics by electroporation. The electroporation protocol was adapted from Usman et al. (2018) (40). Exosomes were incorporated with 400 pmol of custom miR-451a mirVana miRNA Mimic (Life Technologies, cat # 4464066, Assay ID: MC10286), or let-7i-5p mirVana miRNA Mimic (Life Technologies, cat # 4464066, Assay ID: MC10211), or negative control #1 mirVana miRNA Mimic (Life Technologies, cat # 4464058). OptiMEM was used to dilute exosomes for electroporation. Exosomes were electroporated using two pulses at 400V twice with 100µL at a time. Then 1mM of EDTA exosome solution was added, to dissolve RNA aggregates not incorporated in exosomes and left 15 minutes at room temperature. Exosome solution was then centrifuged at 5,000 x g for 5 minutes at 4°C. Supernatant was collected and purified using Sephadex G75 column. The void was collected and stored at -20°C until used for cell treatment.

### hiPSC culture

Human induced pluripotent stem cells from a healthy individual (WT-hiPSCs) were purchased from ThermoFisher Scientific (cat # A18945). Sickle cell anemia donor SCA-hiPSCs (cat # WB66718) were obtained from the Wisconsin International Stem Cell Bank (WISCB). HiPSCs were cultured under feeder-free conditions in dishes coated with Geltrex (Fisher Scientific, cat # A1413302). HiPSCs were negative for mycoplasma, passaged using Versene (Fisher Scientific, cat # 15040066), cultured in StemFlex Medium (Fisher Scientific, cat # A3349401) and incubated at 37°C and 5% CO_2_.

### Heme exposure and HiPSC endothelial differentiation

HiPSCs were differentiated using a modified STEMdiff APEL-Li Vascular differentiation method previously described in Park et al., 2020 (41). Briefly, on day 1, E8 medium was replaced with APEL-2Li medium (StemCell Technologies, cat # 5271) supplemented with Activin A (25 ng/mL, Fisher Scientific, cat # 50398465), VEGF (50 ng/mL, Fisher Scientific, cat #501628212), BMP4 (30 ng/mL, Fisher Scientific, cat #50398981), and CHIR99021 (1.5 μM, Fisher Scientific, cat #NC0984214) for the first 2 days. Subsequently, APEL-Li supplemented with VEGF (50 ng/mL) and SB431542 (10 μM, Fisher Scientific, cat # NC0548146) were used and replaced previous differentiation medium every 2 days until cell population reached maximum expression of CD31+ percentage as determined by Flow Cytometry analysis. CD31+ ECs were isolated using CD31+ Dynabeads (Life Technologies, cat # 11155D). ECs were expanded on fibronectin coated plates in Endothelial Growth Medium-2 (Lonza, cat # CC-3162) for at least 7 to 9 days. HiPSC-ECs at 60% confluency were treated with 30 µM of heme (Sigma-Aldrich, Inc., Saint Louis MO, cat # H9039) or volume-equivalent of vehicle (DMSO, ThermoFisher, cat # 85190) for 24 hours.

### Formulation and nanoparticle tracking analysis of liposomes

Synthetically derived PEGylated liposomes containing either mimic miRNAs were formulated in the Dr. J.L. Hood lab at the University of Louisville, using the same percentage lipid components, based on previously established methods (42). Briefly, a lipid commixture including 64.89 mol% lecithin (, Avanti Polar Lipids Inc., Alabaster AL, cat # 850705P-25mg), 32 mol% cholesterol (Sigma-Aldrich, cat # NC9138103), 65.99 mol% phosphatidylcholine (Avanti, cat # 840051P), 1 mol% 16:0 phosphatidylethanolamine(Avanti, cat # 850705P), 1 mol% 1,2-distearoyl-sn-glycero-3-phosphoethanolamine-N-[carboxy(polyethylene glycol (PEG))-2000] (Avanti, cat # 880135P), and 0.01 mol% 1,1′-Dioctadecyl-3,3,3′,3′-tetramethylindocarbocyanine perchlorate (Millipore Sigma, Burlington MA cat # 42364-100MG) was solubilized in chloroform (Sigma-Aldrich, cat # CX1060-1), and dried to a lipid film under continuous vacuum using an IKA RV 10 rotary evaporator. Residual solvent was removed by drying under continuous vacuum. The dry lipid film was resuspended in 1 mL of miRNA mimic supplied by the manufacturer in molecular grade water. Following resuspension, liposomes were generated using a QSonica Q500 sonicator. Post liposome formulation, liposome sizes were determined by Dr. Ming Bo Huang at the MSM’s Microvesicles Core using a NanoSight LM10-HSBF Nanoparticle Characterization System (Malvern Panalytical Ltd. Malvern UK). Zeta potential analyses were determined by the J.L. Hood lab at the University of Louisville using a PMX 120 ZetaView^®^ (Particle Metrix Inc., Ammersee Germany) Nanoparticle Tracking Analyzer.

### RNA isolation and reverse transcription quantitative PCR

Total miRNA was isolated from exosomes using Total Exosome RNA/Protein Isolation kit (Invitrogen cat # 4478545) following manufactures protocol. Concentrated exosome pellets were resuspended in 200 µL of 1X PBS. Total RNA was isolated from exosomes following manufactures protocol. RNA was eluted in a total of 50 µL of Elution Solution. RNA concentration and purity were determined using the Thermo Scientific NanoDrop 1000 Spectrophotometer. Samples were stored at -80°C until further analysis. MiRNA was reverse-transcribed using Qiagen’s miRCURY LNT RT kit (cat # 339340) and PCR was done using miRCURY LNA SYBR Green PCR Kit (cat # 33936). RT-qPCR was preformed using the Bio-Rad CFX Real-Time PCR Detection Systems. Primers for miR-451a (YP02119305), let-7i-5p (YP00204394), U6 (YP02119464) snRNA (internal control) (cat # 339306) and miR-103-3p (internal control) (cat # 339306) were purchased from Qiagen.

Total RNA was extracted from treated hiPSC-ECs using TRIzol LS (ThermoFisher cat # 10296028) following manufactures protocol. cDNA was synthesized from the RNA collected using iScript cDNA Synthesis kit (Bio-Rad cat # 1708890). cDNA was mixed with SsoAdvanced Universal SYBR Green Supermix (BioRad cat# 1725271) and primers. RT-qPCRs were preformed using Bio-Rad CFX Real-Time PCR Detection Systems. ΔΔCt analysis was completed to determine relative fold expressions. qPCR primers are listed in **Supplementary Table 1** (43, 44).

### Cytokine multiplex assay

2 x 10^5^ cells per well were plated in a 6 well dish and allowed to grow to 80% confluency (about 1x10^6^ cells). Supernatant from hiPSC-EC cell culture was collected after 24 hrs incubation with heme and centrifuged at 10,000 x g for 5 minutes at 4°C. Supernatant cytokine concentrations were determined using the Bio-Plex Pro Human Cytokine Group I Panel (Bio-Rad cat # M500KCAF0Y) which targeted 27 cytokines: Interleukin (IL)-1B, IL-1RA, IL-2, IL-4, Il-5, IL-6, IL-7, Il-8, IL-9, IL-10, IL-12, IL-13, IL-15, IL-17A, CXCL10, CCL2, MIP-1α/CCL3, MIP-1b, PDGF-BB, RANTES, TNF-a, VEGF, FGF basic, Eotaxin, G-CSF, GM-CSF, and IFN-g. The plates were analyzed according to manufacturer’s specifications using a multiplexed microsphere cytokine immunoassay procedure (Magpix, Millipore # 40-072). To account for cell number disparity in treatment group, cytokine ratio was normalized to FGF basic.

### NanoSight analysis

All NanoSight analysis was performed in solution through the technical support of Dr. Ming Bo Huang at the Microvesicle Core of Morehouse School of Medicine (MSM), Atlanta, GA, USA. Briefly, EVs were analyzed using Nanoparticle Tracking Analysis (NTA 2.3) (Malvern Panalytical) at a 1:1000 dilution in PBS. Analysis Settings: frames processed: 749 out of 749, frames per second: 24.99, calibration 190 nm/pixel, threshold 6, temperature: 21.06°C, viscocity: 0.97 cP.

### Electron microscopy

Exosomes isolated from RBCs were visualized *via* negative stain electron microscopy through the service of Robert P. Apkarian Integrated Electron Microscopy Core at Emory University, Atlanta, GA, USA.

### Light phase contrast imaging

Phase Contrast Images were taken using 10x Ph1 Objective on an Olympus BX41 microscope (Olympus America, Center Valley, PA).

### Flow cytometry

For flow cytometry analysis of endothelial cells (ECs), cells were washed once in PBS, and enzymatically digested with 0.05% trypsin-EDTA (5 min at 37°C) and neutralized with knockout serum. Cells were centrifuged (300 x g for 5 min at room temperature) and resuspended in staining buffer (PBS + 0.05% BSA). Single cell suspensions (<1x10^6^ cells in 100 µL per tube) were incubated for 20 min on ice with 1µL of mouse anti-human CD31-APC (eBiosciences cat# 17-0319-42). Cells were washed with 3 mL of PBS, centrifuged (300 x g for 5 min at room temperature), and resuspended in 300 µL of staining buffer prior to acquisition. Viable cells were analyzed (1,000 events acquired for each sample) using the Guava EasyCyte HT flow cytometer and Guava ExpressPro software (Luminex) (EMD Millipore, Billerica, MA, USA) at the MSM’s RCMI core facility.

### Immunostaining

Endothelial cells were fixed for 10 min using 1% paraformaldehyde in PBS at room temperature. For immunofluorescent staining of chambered slides, fixed cells were blocked for non-specific staining and permeabilized using a blocking solution consisting of PBS, 5% BSA (Sigma, cat# A9418) and 0.05% Tween 20 (Sigma, cat# P1379). Samples were incubated overnight at 4°C with goat anti-human VE-Cadherin (RD Systems, cat# AF938-SP) diluted (5 µg/mL) in blocking solution. ECs were washed with PBS and incubated for 1h at room temperature with donkey anti-goat Alexa Fluor 488 secondary antibody (Fisher Scientific, cat# PIA32814TR). Cells were washed three times, mounted with Prolong Gold anti-fade mounting reagent with DAPI (ThermoFisher, cat# P36931), and incubated overnight.

### Statistics

Statistical analysis was completed using GraphPad Prism version 9.1.0 for Mac (GraphPad Software, La Jolla California) unless otherwise stated, and checked for normality using D’Agostino & Pearson normality test. ΔΔCTs of the expressions of miR-451a and let-7i-5p were correlated with differential blood counts using Pearson correlation. One-way ANOVA and Tukey’s multiple comparison test, comparing more than two groups, or an unpaired t-test, comparing two groups, were used to assess for significance. Paired t-test was used comparing qPCR liposomal treatment conditions. The principal component analysis (PCA) was performed using Past version 4.10 for Windows (Past, Oyind Hammer, USA). Heatmap and dendrogram clustering analysis was completed using R Programming version 4.2.0 (Comprehensive R Archive Network, R Foundation, USA). A significance P value of <0.05 was set for all analysis.

## Results

### Exosomal miR-451a and let-7i-5p levels correlate with Hb levels, white blood cells and platelets counts in Pf infected individuals

We recently reported a population study observing various levels of exosomal miR-451a and let-7i-5p in individuals based on hemoglobin sickle cell genotypes (18). Here, we aimed to further assess data gathered from this study to gauge the extent to which miR-451a and let-7i-5p mediate malaria pathogenesis. A hematological summary of participants included in the study can be found in **Supplemental Table 2**.

Pearson correlation analysis was used to compare each miRNA vs WBC count, Hb, PLT count for malaria positive participants regardless of Hb sickle genotypes (HbAA, HbAS, HbSS, HbAC, HbSC). Of the comparisons done there was a significant correlation for exosomal miR-451a (R^2 =^ 0.12, P<0.01) and let-7i-5p (R^2 =^ 0.07, P<0.05) with Hb levels for malaria positive participants (**Figure 1A**). Also, for malaria positive participants, there was a significant correlation between exosomal miR-451a and WBC (R^2 =^ 0.13, P<0.01) and PLT (R^2 =^ 0.27, P<0.0001) (**Supplemental Figures 1A, B**). These results suggest that both miRNAs may have implications in malaria pathophysiology.

### Exosomal miR-451a and let-7i-5p treatment reduce Pf parasite count

To determine if iRBC produce more exosomal miR-451a and let-7i-5p, we infected RBCs with *Pf* NF54E *in vitro* and collected culture media. Exosomes were collected followed by miRNA extraction, and RT-qPCR analysis. In concurrence with previous findings, we observed no significant differences in exosomal miR-451a produced by RBCs after *Pf* infection of *in vitro* cultures with parasitemia ranging from 0.6% to 6.1% (**Figure 1B**). Likewise, we also found no significant difference in exosomal let-7i-5p after RBC infection (**Figure 1C**).

To understand the physiological significance of exosomal miRNA on *Pf* viability, 1% parasitemia iRBCs (*Pf.* 3D7) were treated with 8.25 x 10^11^ exosomes containing miR-451a, let-7i-5p mimic, no mimic (exosome only), or with no exosome treatment for 24 hrs. Homogenous exosome size of 44 nm was confirmed by NanoSight analysis and exosome were visualized by electron microscopy (**Supplemental Figure 1C**; **Figure 2D**). Exosome contents of the miRNA’s mimics were verified by RT-qPCRs using U6 snRNA and mir-103-3p as internal controls; as both controls are constitutively found in exosomes (**Supplemental Figures 1E, F**) (45). These three methods of analaysis provide us with confidence that exosomes have been isolated and successfully electroporated. iRBCs treated with exosomes containing mimic miR-451a or let-7i-5p at a 1:5 dilution (suspended exosome:cell media) for 24 hrs reduced parasitemia by 68% (P<0.05) and 80% (P<0.0001) respectively (**Figures 1E, F**). As expected, parasitemia did not change with no treatment after 24 hrs (n=3) but consistently showed parasitemia of 0.2% when treated with let-7i-5p loaded exosomes (n=3) (**Figure 1F**).

### HiPSC-endothelial cells exposed to heme express the molecular signature associated with malaria or SCA pathophysiology

Utilizing hiPSCs allow for the derivation of multiple tissue types from various genetic backgrounds. This capability creates the opportunity to model population studies on a smaller scale while also elucidating complex pathogenesis. We aimed to establish an hiPSC-EC heme inflammation induced model in order to further elucidate the molecular significance of miR-451a and let-7i-5p. A previously reported optimized vascular differentiation system (41) was employed producing verified CD31+ cells by day 7 of differentiation (**Supplemental Figures 2A-C**). Isolated CD31+ cells showed homogenous expression of VE-Cadherin (CD144), a markers of endothelial cell commitment, after 1 week of cell expansion *in vitro* (**Supplemental Figure 2D**). Previous reports have shown treatment of various cell-types with free-heme could mimic cellular responses to hemolysis mediated inflammation and malaria pathology. Heme concentration was experimentally determined by cell viability assay after 24 hrs treatment (**Supplemental Figure 2E**). Following heme treatment, hiPSC-ECs total lysate of cells and exosomes from culture media were collected, and miRNA levels were analyzed. Heme induced a 6000 (P<0.001) fold increase of exosomal let-7i-5p in WT-hiPSC derived ECs (WT-ECs) (**Figure 2F**), and 4.6 folds in SCA-hiPSC-ECs) (**Figure 2H**). Interestingly, miR-451a exosomal levels as well as cellular miR-451a and let-7i-5p transcripts were not significantly changed in hiPSC-ECs from either Hb backgrounds (**Figures 2A–D**). These findings suggest that heme induces a specific molecular cascade leading to increased let-7i-5p exosomal signaling.

To further characterize the molecular response of hiPSC-ECs to free-heme treatment, we employed a multiplexed cytokine assay of cell media supernatant after 24 hrs of treatment. Heatmap analysis and dendrogram clustering of untreated hiPSC-ECs, hiPSC-ECs treated with DMSO (vehicle), and heme treatment, showed differential inflammatory expression landscape between treatment groups. The most significant cytokines that were differentially expressed included G-CSF, IL-8, and CCL2 (MCP1) (**Supplemental Figure 3**). Principal component analysis (PCA) of treatment groups showed differential clustering could be achieved *via* analysis of the multiplex inflammatory cytokine profile. Untreated and vehicle treated hiPSC-ECs clustered more closely along Principal Component 1—composed of IL-8, G-CSF, MCP-1(CCL2)— while heme treatment groups clustered separately (**Figure 2I**). Interestingly, we also observed differential clustering not only by treatment group, but also by Hb sickle genotype. SCA-hiPSC-ECs clustered separately from WT-hiPSC-EC treated groups. Specifically, heme treated SCA-hiPSC-ECs showed an attenuated inflammatory response compared to WT-hiPSC-ECs resulting in closer clustering to the untreated and vehicle treatment groups (**Figure 2I**, **Supplemental Figure 3**). Based on these findings we explored whether cytokine analysis of these hiPSC-ECs from different Hb sickle genotypes could recapitulate the differential cytokine expression profile previously observed in our population study (39) of malaria infected patients.

We previously reported HbSS patients infected with *Pf* had significantly decreased serum levels of IL-8 and the inflammatory ratio of CCL2:TNF compared to malaria infected HbAA patients (39). These cytokine differences were not seen in cytokine analysis between uninfected HbAA and HbSS patients, suggesting these differences were a result of differential response to malaria infection (39). Analysis of IL-8 levels in the supernatant of heme treated SCA-hiPSC-ECs and WT-hiPSC-ECs also showed significantly lower secretion of IL-8 (P<0.01) from heme treated SCA-hiPSC-ECs (**Figure 2J**). We did not observe a significant decrease in the inflammatory ratio of CCL2:TNF (P=0.1456) between heme treated SCA-hiPSC-ECs and WT-hiPSC-ECs, despite a trend (**Figure 2K**). These findings suggest that our hiPSC-EC model can recapitulate some aspects of the inflammatory response seen in HbAA and HbSS patients.

### MiR-451a and let-7i-5p-loaded liposomes mitigate heme-induced inflammation

To better understand the role of miRNAs in heme-induced inflammation we implemented the use of miRNAs-loaded in PEGylated liposome for cellular treatment. Liposomes, unlike exosomes, are synthetically produced which decreases the potential of extraneous miRNA contaminants (46). Successful derivation of liposomes was confirmed by NanoSight analysis showing a peak diameter of 46 with a standard deviation of 21 nm (**Supplemental Figures 4A, B**). Additionally, zeta potentials of liposomes showed similar distributions, indicating no difference in liposomal stability or aggregation potential. Median zeta potential of liposomes loaded with either negative control mimic, miR-451a, or let-7i-5p was -47.27 ±.56 mV, -47.23±.83 mV, and -46.76±.75 mV respectively (**Supplemental Figure 4C**). The transfer of miRNAs from liposomes to ECs was confirmed by RT-qPCR. In ECs incubated with let-7i-5p loaded liposomes for 6 hours, let-7i-5p expression increased 1.67-fold (p<0.05) (**Supplemental Figure 4D**). Also after 6 hrs of treatment with miR-451a loaded liposomes, ECs expressed 466-fold (P<0.001) higher levels of miR-451a than no-treatment (**Supplemental Figure 4D**). Both ΔΔCt measurements were made using miR-103a-3p as an internal control.

**Figure 3:**
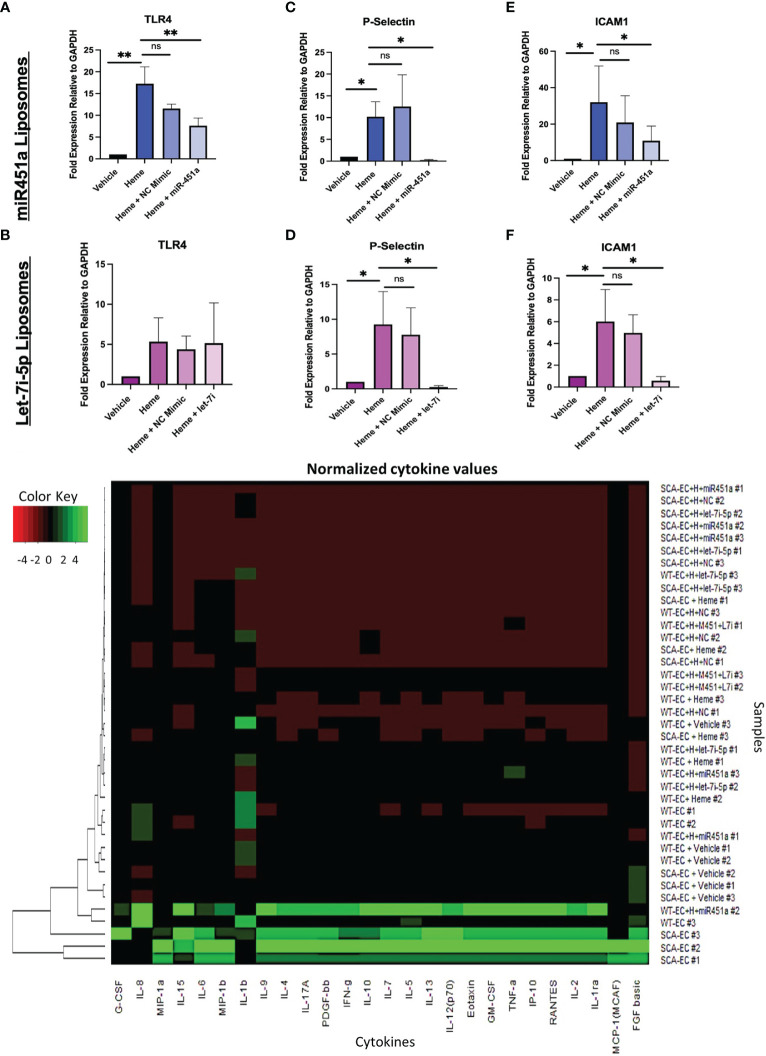
Liposome Treatment of Endothelial Cells **(A–F)** RT-qPCR of total mRNA expression of TLR4, ICAM1, P-Selectin in hWT-ECs treated with indicated miRNA-loaded liposome for 24 hrs. Heme + NC Mimic = hWT-ECs treated with liposomes loaded with negative control nucleic acid mimic. Heme + miR-451a = hWT-ECs treated with liposomes loaded with miR-451a nucleic acid mimic. Heme + let-7i = hWT-ECs treated with liposomes loaded with let-7i-5p nucleic acid mimic. ANOVA was completed followed by individual paired t-test of heme treatment group with other treatment groups for statistical analysis (n=3). No significant difference was found in TLR4 mRNA expression after let-7i-5p liposomal treatment of hWT-ECs. **(G)** Heatmap of Normalized cytokine concentration as determined through cytokine multiplex assay of hWT-ECs and heme treated SCA-ECs after 24 hr. treatment of miR-451a, let-7i-5p, or both miR-451a and let-7i-5p loaded liposomes for 24 hrs (n=3). ns indicates no significant difference, * indicates p < 0.05, ** indicates p < 0.01 for all bar graphs.

TLR4 signaling has been implicated in the pathogenesis of severe malaria including cerebral malaria, and free-heme has been shown to induce TLR4 expression in endothelial cells (43, 47). We hypothesized that miRNA-loaded extracellular vesicles would mitigate TLR4 in our hiPSC-EC model, as miR-451a has been shown to decrease the mRNA levels of TLR4 in murine cerebral ischemia models (48–50). The concentration of miRNA-loaded liposome treatment was experimentally determined based on attenuated TLR4 mRNA transcription. MiR-451a or negative control mimic (NC mimic) loaded liposomes were added to heme treated WT-ECs (hWT-ECs) ranging from 5 pg/mL (1:100 dilution) to 5x10^-2^ pg/mL (1:10,000 dilution) for 24 hrs. Twenty-four-hour heme treatment of WT-ECs caused a 17.25- fold increase (P<0.01) in TLR4 mRNA expression (**Supplemental Figure 4F**). These results are similar to previously reported (43) findings of heme treatment on immortalized and primary EC, further confirming the validity of our hiPSC-EC model. TLR4 mRNA expression was decreased after treatment with miR-451a liposomes and inversely correlated with the concentration of miR-451a liposome added (**Supplemental Figure 4F**). WT-hiPSC-ECs treated with 5pg/mL significantly reduced TLR4 mRNA expression by 2.3-fold (P<0.01) (**Figure 3A**). TLR4 mRNA expression was not significantly changed by treatment with let-7i-5p loaded liposomes in hWT-EC (**Figure 3B**). We then evaluated whether treatment with miRNA-loaded liposomes affected downstream targets of TLR4. For that we evaluated mRNA expression of the cell surface ligands P-selectin and the Intercellular Adhesion Molecule 1 (ICAM1), as both have been shown to directly bind to the *Pf* specific protein *Pf* erythrocyte membrane protein 1 (Pfemp1) (5, 51, 52). hWT-EC P-selectin mRNA expression was significantly reduced by 43-fold (P <0.05) and 38-fold (P<0.05) after 24 hrs treatment of miR-451a and let-7i-5p-loaded liposomes respectively (**Figures 3C, D**). Likewise, hWT-EC ICAM1 mRNA expression was reduced by 2.97-fold (P<0.05) and 10.29-fold (P<0.05) after miR-451a and let-7i-5p-liposome treatment respectively (**Figures 3E, F**).

Using multiplex cytokine analysis of hWT-EC supernatant treated with liposomes, we analyzed whether miR-451a or let-7i-5p loaded liposomal treatment would alter the inflammatory profile observed after heme treatment (**Figure 3G**). hWT-ECs treated with miR-451a liposomes significantly decreased the release of GM-CSF (p<0.05) (**Supplemental Figure 5A**). hWT-ECs treated with let-7i-5p liposomes showed significant reduction in the abundance of GM-CSF, IFN-gamma, PDGF-bb, TNF-alpha cytokines levels (**Supplemental Figures 5A-D**). When hWT-ECs were treated with both miR-451a and let-7i-5p loaded liposomes we observed a decreased levels of CXCL10, IFN-gamma, IL-7, G-CSF, PDGF-bb, IL-1ra, and TNF-alpha in cell supernatant (**Supplemental Figure 5**). These findings suggest that both miR-451a and let-7i-5p have anti-inflammatory roles that are distinguishable from each other, but together may synergistically attenuate the inflammatory cascade induced by heme treatment. We did not find significant reduction in cytokine protein expression in heme-treated SCA-ECs (hSCA-ECs) conditions with either miR-451a or let-7i-5p loaded liposomes (**Supplemental Figures 5L-P**).

## Discussion

The study of exosomal miRNAs from patient samples as potential biomarkers of malaria pathophysiology is a growing field with implications to better stratify *Pf* infection and disease severity (15–17). We and others have shown both exosomal miR-451a and let-7i-5p could be used for such an endeavor as well as the further stratification of these exosomal miRNA by hemoglobin sickle genotype (18, 19, 22). In this study we provide novel insights into the molecular roles of EV-loaded miR-451a and let-7i-5p in heme-induced inflammation. We have shown that the levels of both miR-451a and let-7i-5p loaded exosomes directly correlate with Hb levels in malaria patients suggesting a physiological role for these specific miRNA-loaded exosomal release (**Figure 1A**, **Supplemental Figures 1B, C**). We thus sought to trace which cell types were responsible for miR-451a and let-7i-5p exosomal release (**Figures 1B-C**, **Figures 2E-H**). To our surprise we did not find an increase in exosome levels of either mir-451a or let-7i-5p after RBC infection with *Pf*, in contrast to the already mentioned population studies showing increased miR-451a and let-7i-5p exosome levels in infected patients (15–17). Increased exosomal miR-451a and let-7i-5p levels in malaria patients should be therefore not a direct result of *Pf* infection of RBCs caused by either iRBC exosome release or lysis. However, we are the first to report that exosomal miR-451a and let-7i-5p treatment of *Pf-*infected RBCs inhibit the viability of *Pf in vitro* (**Figures 1E, F**). It was previously reported that both miRNAs anneal with *Pf* mRNA inhibiting translation and leading to reduced *Pf* growth after miRNA transfection (19). We are able to mimic these results by a different mechanism of miRNA delivery. The experiments also showed some effect on parasitemia when treated with un-electroporated exosomes alone (**Figures 1E, F**), although it was not significant (p>0.05). Since exosomes collected from plasma are not empty vacuoles, they may contain innate miRNAs or other biomolecules that could have effects on Pf growth. Even so, let-7i-5p loaded EVs showed significant reductions in parasitemia when compared to un-electroporated EV controls (**Figure 1F**).

Using hiPSC-EC from WT and SCA Hb sickle genotypes we explored the inflammatory responses induced by the presence of free-heme in culture. Previous reports have shown that heme inhibits angiogenesis as shown by a decrease in CD31 expression. This is most likely mediated by TLR4, as heme activates the TLR4 receptor and TLR4 signaling drastically inhibits microcapillary structural integrity (53–56).

**Figure 4:**
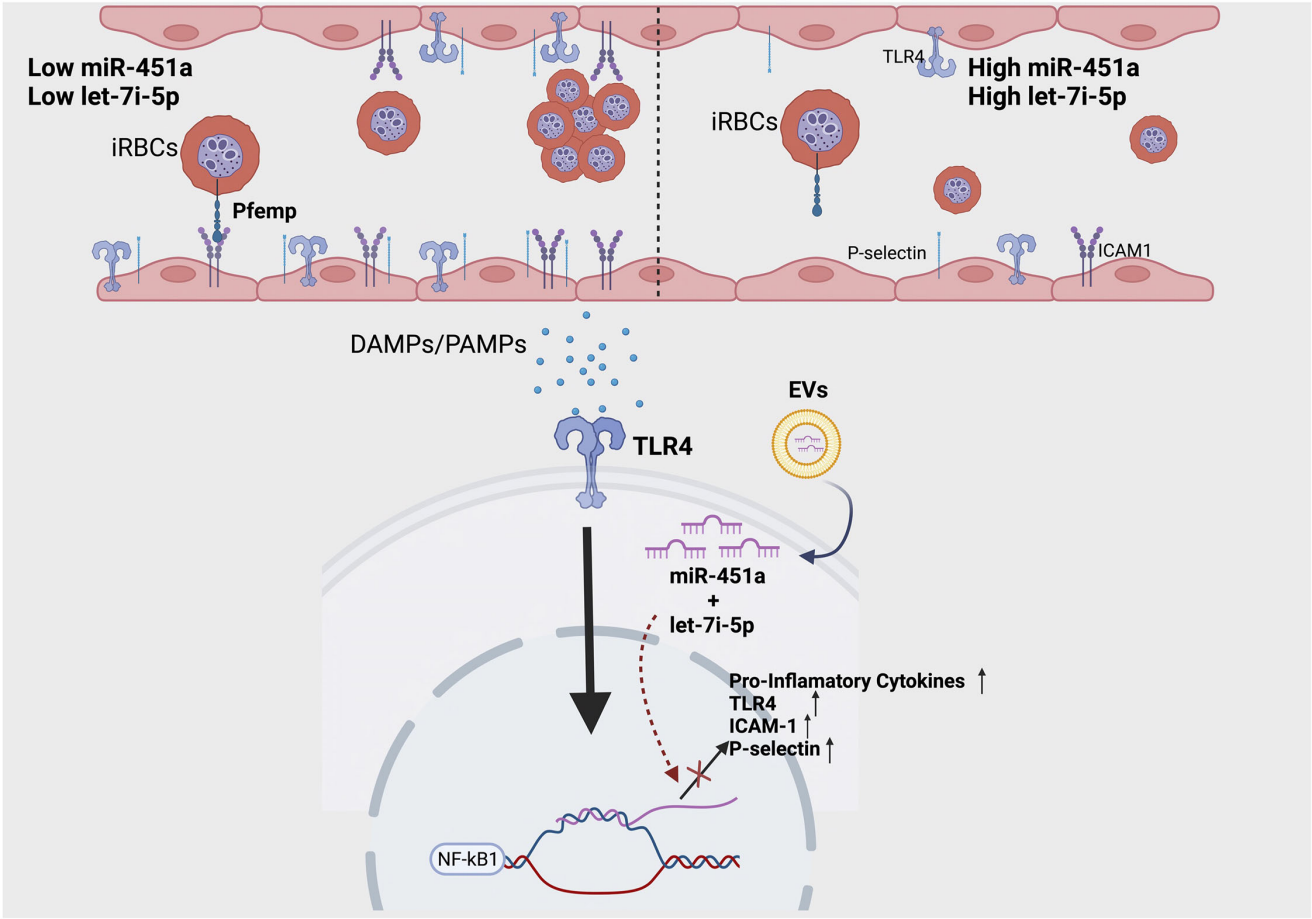
Proposed model of molecular significance of miR-451a and let-7i-5p loaded EVs in hemolytic induced inflammation *via* malaria infection. Patients with low circulating miR-451a or let-7i-5p loaded EVs have increased inflammatory cytokines release and increased expression of adhesion molecules. This increases the likelihood of iRBC stasis *via* the binding of malaria-specific ligand Pfempf1. Damage Associated Molecular Patterns (DAMPs), such as free-heme, and Pathogen Associated Molecular Patterns (PAMPs) activate TLR4 receptor inducing NFκК-β transcription factor signaling. MiR-451a and let-7i-5p inhibits the TLR4/NFK-β inflammatory cascade by decreasing mRNA transcription of TLR4, endothelial ligands, or pro-inflammatory cytokines. Image was created with BioRender.com.

We report the novel finding of a significant increase of exosomal let-7i-5p from ECs after exposure to heme; and no difference in exosomal miR451a release (**Figures 2E-H**). Based on let-7 miRNAs’ well-established role in inducing erythropoiesis (56), as well as the significant positive correlation between exosomal let-7i-5p and Hb abundance in positive malaria patients, our data suggest a novel feedback loop in which anemic crisis due to RBC lysis causes the release of EC derived-exosomal let-7i-5p as a compensatory mechanism to induce erythropoiesis (**Figures 2E-H**). Furthermore, using our hiPSC-EC model, we found distinct inflammatory cytokine profiles based on Hb sickle genotypes (**Figure 2I**). Similar differences in cytokine expression between WT and SCA derived ECs, previously reported in our population study (39), were also observed *in vitro* (**Figures 2J, K**). Though not previously reported in the context of SCA-iPSCs, we note that this finding is consistent with other reports using hiPSCs derived from patient samples of chronic inflammatory disorders (41, 57). As such, we do speculate the possibility that our SCA-hiPSCs retained their chronic inflammatory characteristics and thus could be used for further disease modeling (58, 59). Based on the remarkable anti-inflammatory effects of miR-451a and let-7i-5p-loaded liposomes on heme-treated hiPSC-ECs, our data, like others (60), strongly suggest an important role of small extracellular vesicle in modulating inflammatory processes associated with pathogens such as *Plasmodium* (**Figure 3**). Specifically, in the context of malaria we propose a model in which malaria patients with low circulating miR-451a and let-7i-5p-loaded EVs exhibit an increased inflammatory response to free-heme release associated with infected RBC lysis, the increased abundance of Damage Associated Molecular Patterns (DAMPs)—such as free-heme—and Pathogen Associated Molecular Patterns (PAMPs) **(** **Figure 4**). This could result in increased expression of intrinsic *Pfemp1* ligands (i.e., ICAM1 and P-selectin) mediated by TLR4 signaling on endothelial cells and iRBC stasis (52) (**Figure 4**). Infected-RBCs attachment to endothelial cells within the micro-vessels of the brain leads to acute ischemia, decrease integrity of endothelial junctions—and if not mitigated—hemorrhage and death (32). It is thus significant that both miR-451a and let-7i-5p loaded EVs reduced Pfemp1 ligands on endothelial cells (**Figures 3A-F**). MiR-451a and let-7i-5p loaded EVs therefore has pleiotropic roles in malaria pathophysiology including inhibiting *Pf* growth, decreasing the release of pro-inflammatory cytokines, inhibiting the transcription of pathogen-associated ligands, and may also decrease the risk of severe anemic crisis found in severe cases of malaria.

In contrast to the findings of Mantel et al. (22), we did not find a pro-inflammatory role of either miR-451a and let-7i-5p when administered on iPSC-derived endothelial cells. This may be context driven as Mantel et al. (22) administered RBC-derived exosomes loaded with miR-451a which possibly could contain pro-inflammatory ligands or cytokines already within the collected exosomes. Additionally, Mantel et al. (22) did not pre-treat endothelial cells with free heme, thus a different regulatory program may be at play within our model compared to theirs. Even so, our findings of an anti-inflammatory mechanism of miR451-a and let-7 miRNAs through a possible TLR4 inhibition mechanism has been noted in other infectious models (48, 61–63). MiR-451a has been shown to form an anti-sense complex with TLR4 mRNA leading to TLR4 mRNA destruction (48). Additionally, let-7 miRNAs dampen toll-like receptor activity in macrophages and neutrophils through an unclear mechanism of action (61, 62). We also note that co-administration of EV-loaded miR-451a and let-7i-5p led to the significant reduction of the TLR4 dependent release CXCL10 (**Supplemental Figure 5E**), a biomarker of cerebral malaria severity (64). The significance of these findings is bolstered by previous work showing pharmacological reduction of CXCL10 in addition to anti-malarial treatment leading to increased survival of mice in an *in vivo* cerebral malaria model (7). Thus, more studies are required to explore the potential therapeutic use of EV-loaded miR-451a and let-7i-5p to mitigate inflammation in malaria patients using nanoparticle gene therapy technology.

Future studies will explore the regulation of miR-451a and let-7i-5p loaded exosome release in endothelial cells and whether it can be pharmacologically increased. Our findings that increased release of exosomal let-7i-5p does not correlate with increased cellular transcription (**Figures 2F, H**) suggest cellular storage of let-7i-5p in a yet undetermined cellular compartment. Exploring how to pharmacologically release these stores, could help mitigate *Pf* growth and heme-induced inflammation *in vivo*. Additionally, in the future we aim to identify and study the cells responsible, as well as the cellular signal required for miR-451a loaded exosome release. Further studies are required to test other cell types including erythrocytes, macrophages, hepatocytes, and neural tissue in the context of free heme to explore this question. Finally, we hope to expand our iPSC-derived model to produce neural and hepatic tissue in the hopes to gain greater physiological understanding of the impact of circulating miR-451a and let-7i-5p EVs.

## Data availability statement

The original contributions presented in the study are included in the article/**Supplementary Material**. Further inquiries can be directed to the corresponding author.

## Ethics statement

The studies involving human participants were reviewed and approved by Morehouse School of Medicine Korle-bu Teaching Hospital. Written informed consent to participate in this study was provided by the participants’ legal guardian/next of kin.

## Author contributions

AD, JS, JT, and KH designed the study. AD, MW, JS, KH and FB provided support in subject enrollment and sample collection. JT, KH, AB, AD and JH conducted the experiments. JT, KH, AD, and JS analyzed and interpreted the data. JH processed the construction of the miRNA-loaded liposomes. JKS, WT, MDW and AD’s laboratories were used to conduct the experiments. JT and AD wrote the paper. JT, KH, AB, FB, MW, JH, WT, JS, and AD edited and approved the final manuscript. All authors contributed to the article and approved the submitted version.

## Supplementary material

Supplementary Table 2 Cohort hematological characteristics of malaria positive study participants of all Hb genotypes ((HbAA, HbAS, HbSS, HbAC, HbSC) previously described in Harp et al., 2022 (18) with mean and standard deviation (Stdev) for each characteristic. Normal range for white blood cells (WBCs), is 4.5-11 x 10^3^/mm^3^. Normal red blood cells (RBCs) range for women are 4.1-5.1 x 10^6/^μL and men are 4.5-5.9 x 10^6^/μL. Normal range for hemoglobin (Hb) is 13.5 to 17.5 g/dL for men and 12.0 to 15.5 g/dL for women. Normal range for hematocrit (Hct) is 37-52% and for platelets (PLT) is 150-450 x 10^3^/μL. Normal values of Mean Cellular Volume (MCV) is 80-100 μm^3^.

Supplementary Figure 1
**(A)** Pearson correlation between exosomal miR-451a and WBC resulted in R^2 =^ 0.13 and P<0.01. **(B**) Pearson Correlation between exosomal miR-451a and PLT resulted in R^2 =^ 0.27, P<0.0001. **(C)** NanoSight results indicating exosomes are present and correct size. **(D, E)**. RNA extracted from exosomes loaded with let-7i-5p or miR-451a mimics vs exosomes that were not electroporated with any additional RNA (Empty Exosomes) was evaluated using RT-qPCR. Let-7i-5p and miR-451a levels were significantly elevated in exosomes with miRNA mimics compared to empty exosomes as tested with paired t-test statistical analysis (P<0.0001).

Supplementary Figure 2
**(A)** Summary of APEL-2Li Vascular Differentiation Protocol adapted from Park et al. (2020) **(B)** Phase Contrast using 20x objective of undifferentiated (day 1) and differentiated (day 7) and CD31+ sorted cell derived from WT-hiPSC and SCA-hiPSC using the APEL-2Li vascular differentiation protocol. Bar = 100µm. **(C)** Flow cytometry analysis of Day 7 cells of the APEL differentiation protocol stained with CD31-APC antibody. Seven Days post differentiation of hiPSCs, differentiation protocol produced 15.99% CD31+ cells representing putative endothelial cells. **(D)** Immunofluorescence stain for VE-Cadherin on CD31+ sorted endothelial cells expanded for 2 weeks in EGM2 medium. Bar = 100µm. **(E)** Viability assay of heme treated endothelial cells using Trypan Blue Dye. Cell Viability was calculated using automated cell counter (n=3). (P<0.05 (two-tail t-test), ns= not significant).

Supplementary Figure 3 Cluster Dendrogram of normalized cytokine multiplex data representing key differential cytokines between treatment groups.

Supplementary Figure 4
**(A, B)** NanoSight Analysis of synthetically derived liposome nanoparticle loaded with miR-451a mimics nucleotide. Similar findings have been found in let-7i-5p loaded nanoparticles (Data Not Shown). **(C)** Zeta potential distribution analyses of nucleotide mimic liposomes were determined by the J.L. Hood lab at the University of Louisville using a PMX 120 ZetaView^®^ nanoparticle tracking analyzer. No significant differences in average zeta potential between liposome types were determined. **(D, E)** RT-qPCR analysis of let-7i-5p **(D)** and miR-451a **(E)** expression in ECs treated with miR-451a or let-7i-5p loaded liposomes respectively for 6 hrs (n=3). Let-7i-5p expression increased 1.67-fold (p<0.05) **(D)** and miR-451a increased 466-fold (P<0.001) **(E)** compared to no-treatment. A Student-t test was used for statistical analysis of the ΔΔCt relative expression, using miR-103a-3p as an internal control. **(F)** RT-qPCR gene expression analysis of TLR4 in heme treated endothelial cells with annotated dilutions liposomes loaded with negative control mimic or miR-451a mimic (n=3) (ANOVA was first performed followed by multiple Comparison two-tail t-test analysis p<0.01).

Supplementary Figure 5
**(A–P)** Quantitative analysis of cytokine concentration Bio-Plex Pro Human Cytokine Group I Panel (Bio-Rad cat # M500KCAF0Y). Y axis indicates concentration in pg/mL. One-way ANOVA and Tukey’s multiple comparison test performed. (*= P<0.05, **=p<0.01).
