# Supplementary material for: MiR-451a and let-7i-5p loaded extracellular vesicles attenuate heme-induced inflammation in hiPSC-derived endothelial cells
Source: Front Immunol. 2022 Dec 22;13:1082414. doi: 10.3389/fimmu.2022.1082414 (PMC9815029; doi:10.3389/fimmu.2022.1082414)
Supplement: Supplementary file 1 [file DataSheet_1.pdf]

Supplemental Table 1: RT-qPCR primers for gene expression estimation

Name	Sequence	Source
**ICAM1 FWD**	TTCGTGTCCTGTATGGCCC	Thakar et. al (2021) [43]
**ICAM1 REV**	CACATTGGAGTCTGCTGGGA	Thakar et. al (2021) [43]
**P-Selectin FWD**	CCAACCTGCAAAGGCATAGC	Thakar et. al (2021) [43]
**P-Selectin REV**	GCGTTGCAGCCAAAGTAACA	Thakar et. al (2021) [43]
**GAPDH FWD**	TCGGAGTCAACGGATTTGGT	Thakar et. al (2021) [43]
**GAPDH REV**	TTCCCGTTCTCAGCCTTGAC	Thakar et. al (2021) [43]
**TLR4 FWD**	CAGGATGATGTCTGCCTCGC	Dickson-Copelan et. al (2015) [42]
**TLR4 REV**	TTAGGAACCACCTCCACGCAG	Dickson-Copelan et. al (2015) [42]
