# Supplementary material for: MiR-451a and let-7i-5p loaded extracellular vesicles attenuate heme-induced inflammation in hiPSC-derived endothelial cells
Source: Front Immunol. 2022 Dec 22;13:1082414. doi: 10.3389/fimmu.2022.1082414 (PMC9815029; doi:10.3389/fimmu.2022.1082414)
Supplement: Supplementary Table 2 — Cohort hematological characteristics of malaria positive study participants of all Hb genotypes ((HbAA, HbAS, HbSS, HbAC, HbSC) previously described in Harp et al., 2022 (18) with mean and standard deviation (Stdev) for each characteristic. Normal range for white blood cells (WBCs), is 4.5-11 x 103/mm3. Normal red blood cells (RBCs) range for women are 4.1-5.1 x 10^6^/μL and men are 4.5-5.9 x 10^6^/μL. Normal range for hemoglobin (Hb) is 13.5 to 17.5 g/dL for men and 12.0 to 15.5 g/dL for women. Normal range for hematocrit (Hct) is 37-52% and for platelets (PLT) is 150-450 x 103/μL. Normal values of Mean Cellular Volume (MCV) is 80-100 μm3. [file DataSheet_2.pdf]

Supplemental Table 2

	N	Percent	Mean	Stdev
Total Individuals	64			
Male (Sex)	36	56.2%		
Female (Sex)	28	43.8%		
WBC (x 10^3 / mm^3)	63		7.883	5.225
RBC (x 10^6 / µL)	64		3.805	1.114
Hb (g/dL)	64		10.095	2.993
Hct (%)	64		31.016	9.002
MCV (μm^3)	64		82.209	9.641
PLT (x 10^3 / µL)	64		213.828	133.674
