# Supplementary figures and images for: MiR-451a and let-7i-5p loaded extracellular vesicles attenuate heme-induced inflammation in hiPSC-derived endothelial cells
Source: Front Immunol. 2022 Dec 22;13:1082414. doi: 10.3389/fimmu.2022.1082414 (PMC9815029; doi:10.3389/fimmu.2022.1082414)

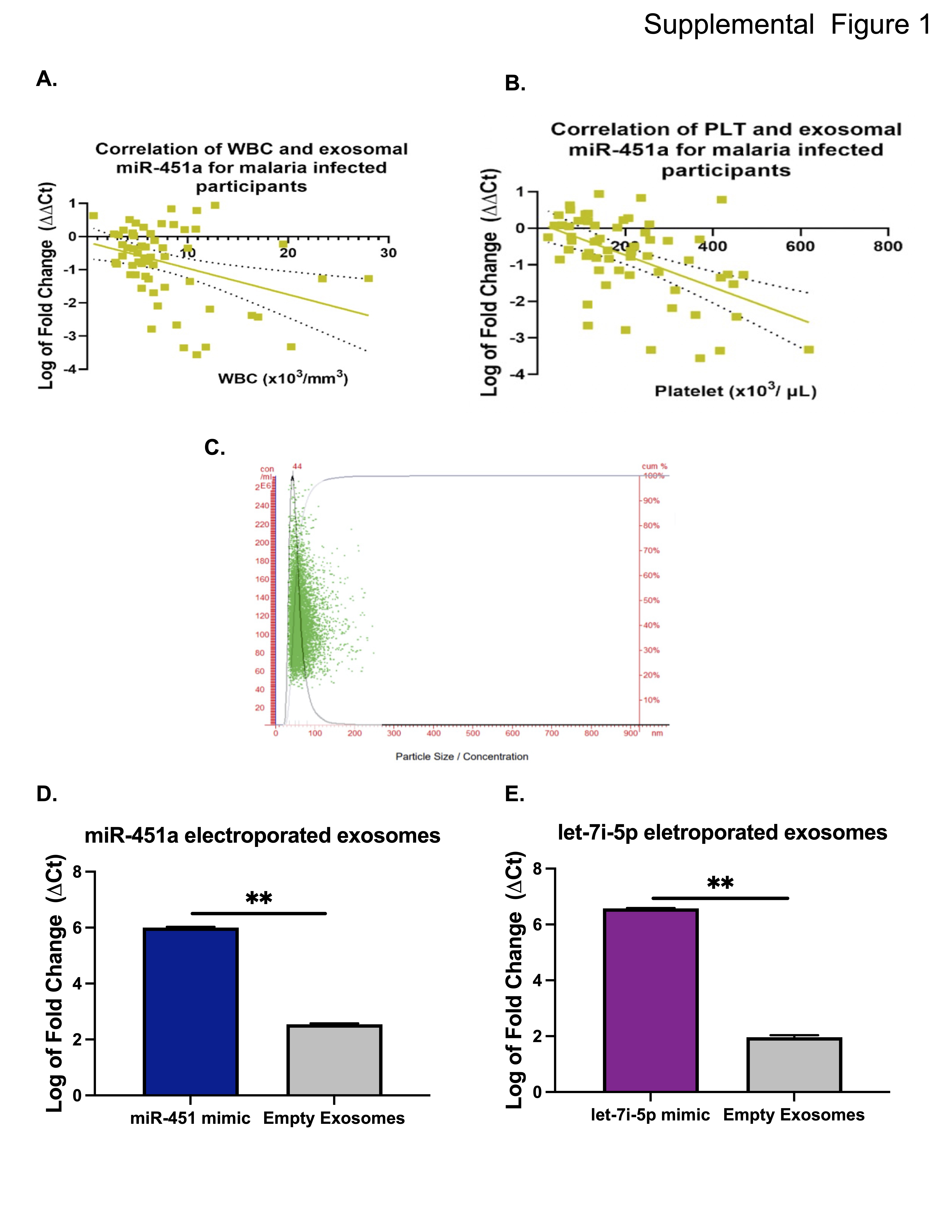

Supplement: Supplementary Figure 1 — (A) Pearson correlation between exosomal miR-451a and WBC resulted in R2 = 0.13 and P<0.01. (B) Pearson Correlation between exosomal miR-451a and PLT resulted in R2 = 0.27, P<0.0001. (C) NanoSight results indicating exosomes are present and correct size. (D, E). RNA extracted from exosomes loaded with let-7i-5p or miR-451a mimics vs exosomes that were not electroporated with any additional RNA (Empty Exosomes) was evaluated using RT-qPCR. Let-7i-5p and miR-451a levels were significantly elevated in exosomes with miRNA mimics compared to empty exosomes as tested with paired t-test statistical analysis (P<0.0001). [file Image_1.jpeg]

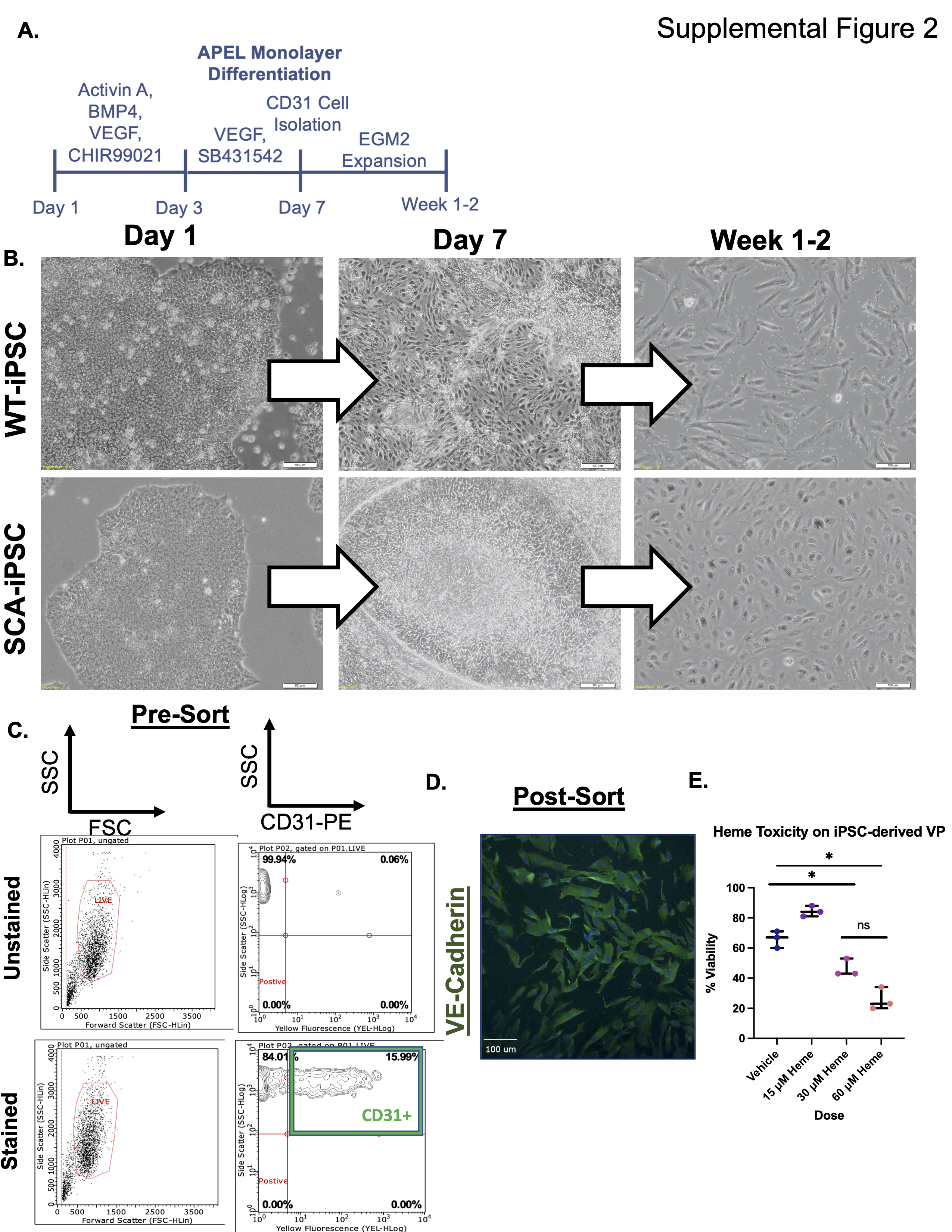

Supplement: Supplementary Figure 2 — (A) Summary of APEL-2Li Vascular Differentiation Protocol adapted from Park et al. (2020) (B) Phase Contrast using 20x objective of undifferentiated (day 1) and differentiated (day 7) and CD31+ sorted cell derived from WT-hiPSC and SCA-hiPSC using the APEL-2Li vascular differentiation protocol. Bar = 100µm. (C) Flow cytometry analysis of Day 7 cells of the APEL differentiation protocol stained with CD31-APC antibody. Seven Days post differentiation of hiPSCs, differentiation protocol produced 15.99% CD31+ cells representing putative endothelial cells. (D) Immunofluorescence stain for VE-Cadherin on CD31+ sorted endothelial cells expanded for 2 weeks in EGM2 medium. Bar = 100µm. (E) Viability assay of heme treated endothelial cells using Trypan Blue Dye. Cell Viability was calculated using automated cell counter (n=3). (P<0.05 (two-tail t-test), ns= not significant). [file Image_2.jpeg]

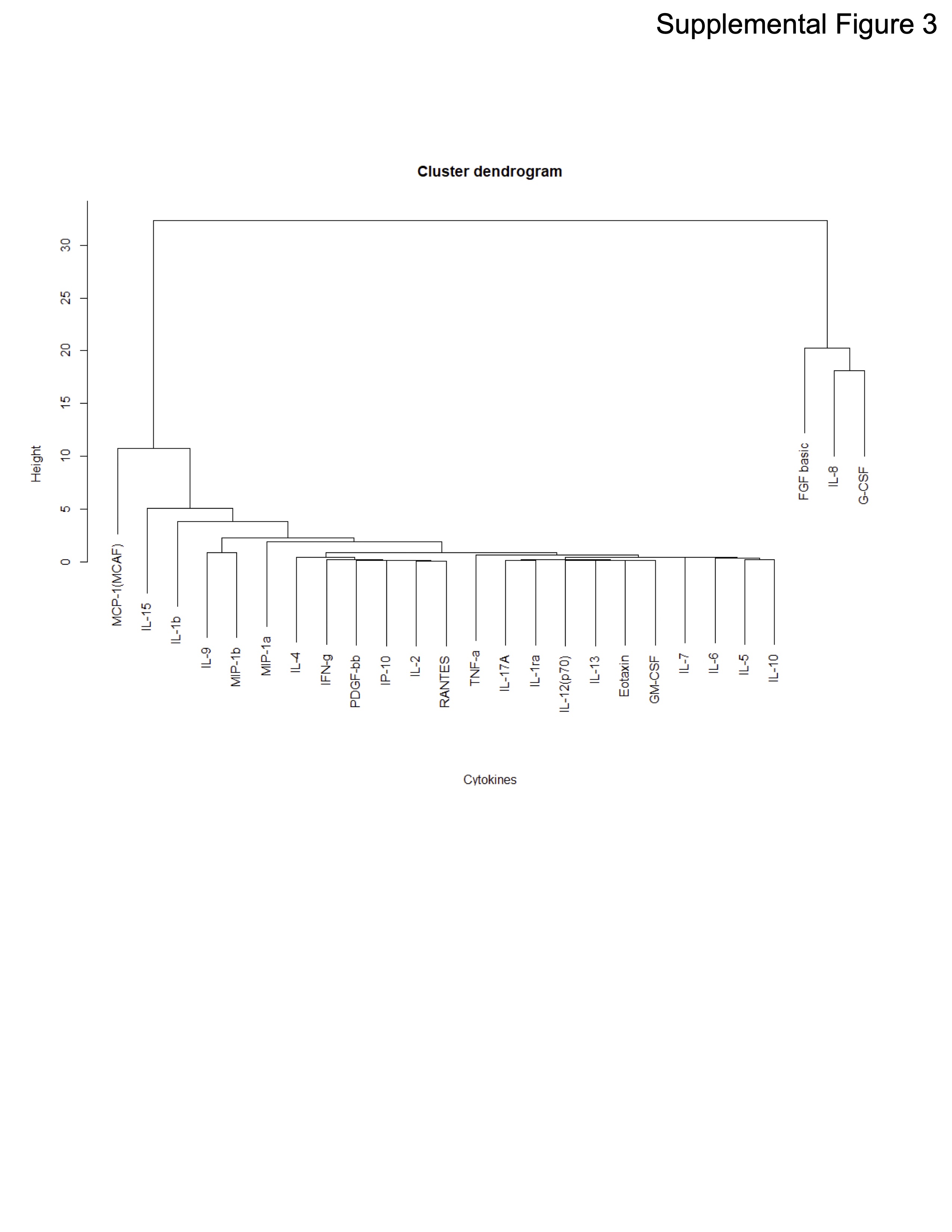

Supplement: Supplementary Figure 3 — Cluster Dendrogram of normalized cytokine multiplex data representing key differential cytokines between treatment groups. [file Image_3.jpeg]

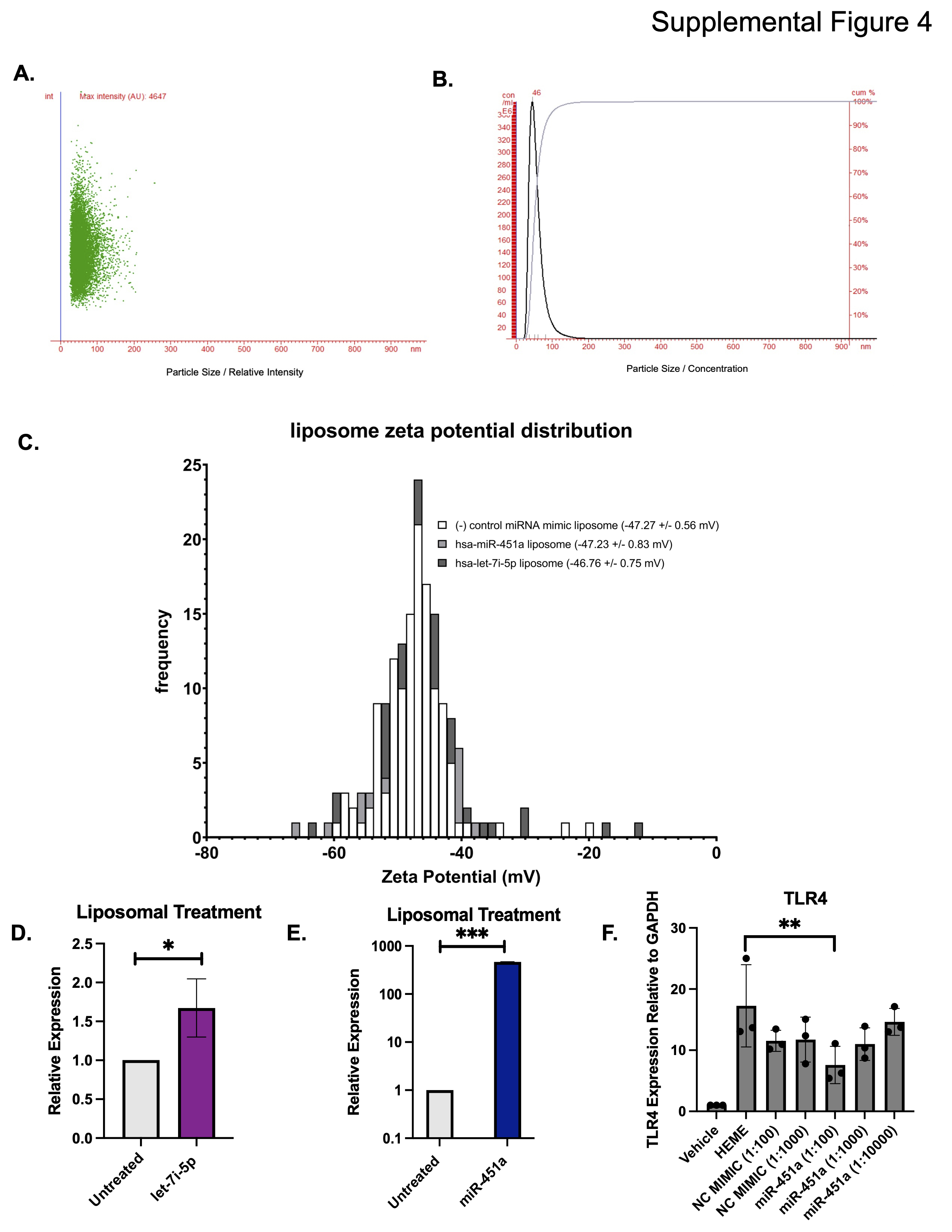

Supplement: Supplementary Figure 4 — (A, B) NanoSight Analysis of synthetically derived liposome nanoparticle loaded with miR-451a mimics nucleotide. Similar findings have been found in let-7i-5p loaded nanoparticles (Data Not Shown). (C) Zeta potential distribution analyses of nucleotide mimic liposomes were determined by the J.L. Hood lab at the University of Louisville using a PMX 120 ZetaView® nanoparticle tracking analyzer. No significant differences in average zeta potential between liposome types were determined. (D, E) RT-qPCR analysis of let-7i-5p (D) and miR-451a (E) expression in ECs treated with miR-451a or let-7i-5p loaded liposomes respectively for 6 hrs (n=3). Let-7i-5p expression increased 1.67-fold (p<0.05) (D) and miR-451a increased 466-fold (P<0.001) (E) compared to no-treatment. A Student-t test was used for statistical analysis of the ΔΔCt relative expression, using miR-103a-3p as an internal control. (F) RT-qPCR gene expression analysis of TLR4 in heme treated endothelial cells with annotated dilutions liposomes loaded with negative control mimic or miR-451a mimic (n=3) (ANOVA was first performed followed by multiple Comparison two-tail t-test analysis p<0.01). [file Image_4.jpeg]

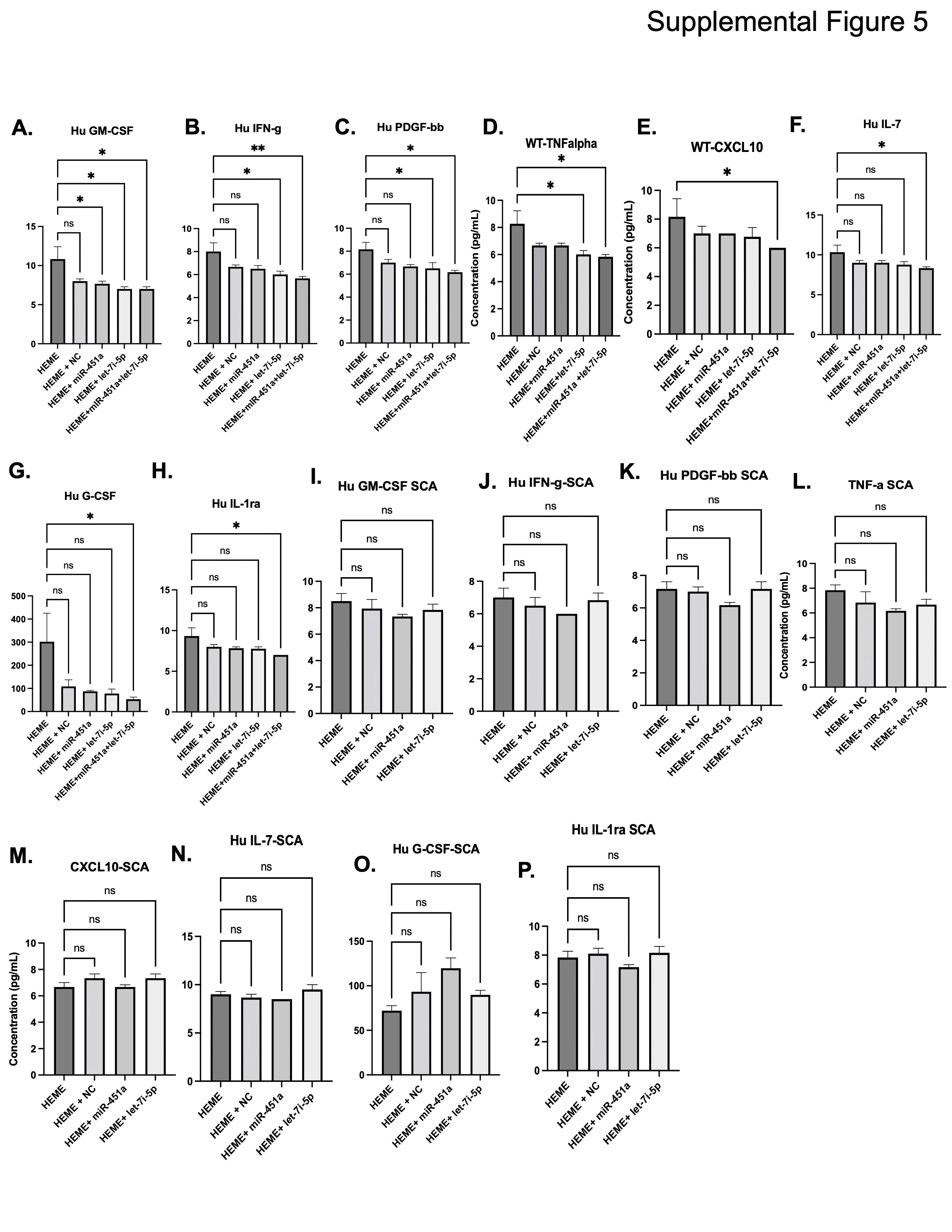

Supplement: Supplementary Figure 5 — (A–P) Quantitative analysis of cytokine concentration Bio-Plex Pro Human Cytokine Group I Panel (Bio-Rad cat # M500KCAF0Y). Y axis indicates concentration in pg/mL. One-way ANOVA and Tukey’s multiple comparison test performed. (*= P<0.05, **=p<0.01). [file Image_5.jpeg]
